# Microbiomics in Collusion with the Nervous System in Carcinogenesis: Diagnosis, Pathogenesis and Treatment

**DOI:** 10.3390/microorganisms9102129

**Published:** 2021-10-11

**Authors:** Rodney Hull, Georgios Lolas, Stylianos Makrogkikas, Lasse D. Jensen, Konstantinos N. Syrigos, George Evangelou, Llewellyn Padayachy, Cyril Egbor, Ravi Mehrotra, Tshepiso Jan Makhafola, Meryl Oyomno, Zodwa Dlamini

**Affiliations:** 1 SAMRC Precision Oncology Research Unit (PORU), Pan African Cancer Research Institute (PACRI), University of Pretoria, Hatfield 0028, South Africa; Rodney.hull@up.ac.za (R.H.); glolas@med.uoa.gr (G.L.); lc.padayachy@up.ac.za (L.P.); cyrilagbor0772@gmail.com (C.E.); ravi.mehrotra@gov.in (R.M.); tshepisomakhafola5@gmail.com (T.J.M.); meryl.oyomno@up.ac.za (M.O.); 2 Department of Medicine, National & Kapodistrian University of Athens, 11527 Athens, Greece; ksyrigos@med.uoa.gr (K.N.S.); grgevangelou@med.uoa.gr (G.E.); 3 FALCONBIO PTE, Ltd., 32 Carpenter Street, SGInnovate, Singapore 059911, Singapore; stelios279@falconbio.org; 4 Division of Cardiovascular Medicine, Department of Health, Medicine and Caring Sciences, Faculty of Medicine, Linköping University, 581 83 Linköping, Sweden; lasse.jensen@liu.se; 5 Department of Neurosurgery, University of Pretoria, Hatfield 0028, South Africa; 6 Centre for Health Innovation and Policy (CHIP) Foundation, Noida 201301, India; 7 Datar Cancer Genetics, Nashik 422010, India; 8 Centre for Quality of Health and Living, Faculty of Health and Environmental Sciences, Central University of Technology, Bloemfontein 9300, South Africa; 9 Department of Surgery, Faculty of Health Sciences, Steve Biko Academic Hospital, University of Pretoria, Pretoria 0007, South Africa

**Keywords:** microbiota, gut–brain axis, metabolites, neurogenesis, neurotransmitters, immunity, carcinogenesis

## Abstract

The influence of the naturally occurring population of microbes on various human diseases has been a topic of much recent interest. Not surprisingly, continuously growing attention is devoted to the existence of a gut brain axis, where the microbiota present in the gut can affect the nervous system through the release of metabolites, stimulation of the immune system, changing the permeability of the blood–brain barrier or activating the vagus nerves. Many of the methods that stimulate the nervous system can also lead to the development of cancer by manipulating pathways associated with the hallmarks of cancer. Moreover, neurogenesis or the creation of new nervous tissue, is associated with the development and progression of cancer in a similar manner as the blood and lymphatic systems. Finally, microbes can secrete neurotransmitters, which can stimulate cancer growth and development. In this review we discuss the latest evidence that support the importance of microbiota and peripheral nerves in cancer development and dissemination.

## 1. Introduction

It has been estimated that the microbiota in a human body consists of approximately 3000 different species of microbes with 40,000,000,000,000 (40 trillion) individual microbial cells. The vast majority of these are gastrointestinal bacteria [1]. One of the first microbial based treatments proposed for cancer was found in the Eber’s Papyrus, written by the Egyptian physician Imhotep around the year 2600 BCE [2]. He proposed that infection of the area affected by the cancer through an open wound covered by a poultice could have therapeutic potential. At that time Germ theory was unknown [3]. This treatment was revived in the 1800s by William Coley who “vaccinated” cancer patients with live or heat-killed Streptococcus and Serratia species [2]. Coley documented 4 cases of sarcoma where the patients were completely cured by erysipelas infection. The medical establishment, however, discounted his treatment as fatal. We now know, erysipelas causes an inflammatory reaction that most probably are negating the sarcoma [4,5]. Coley’s infections with erysipelas showed for the first time that microbes influence the development and progression of cancer. The use of microbe metabolites, supplementing the diets with probiotics or by faecal transfers is now being explored to treat cancers. This new treatment strategy is the result of growing evidence that the microbiota can play an important role in cancer development and progression [6,7].

Each specific region of the human body has its specific microbiome. Those areas with the richest microbiome include the skin, airways, urogenital tract, eyes and gastrointestinal tract [1]. Microbes present in the human body can also interact with the nervous system in various ways. These include via the enteric nervous system, the vagus nerve, microbial metabolites and the immune system [8,9]. Recently, the fact that the microbiome is able to interact with the nervous system and the fact that changes in the microbiota can promote the development of cancer or indeed help prevent or treat cancer, has led to the suggestion that these pathways may be mechanistically connected. Their interaction can be facilitated through the effects of the microbiome on the immune system [10]. A downregulated immune response provides favourable conditions for the development and progression of cancer [11]. A growing body of research demonstrates that changes to the composition of the gastrointestinal microbiota is an initiating factor in numerous neurocognitive conditions, profoundly influencing both CNS immunity and the integrity of the blood–brain barrier (BBB) [12].

It is now well known that the growth and progression of cancer can occur through the support of nerve tissue (neoneurogenesis) in the same way that new blood vessels (angiogenesis), or lymph vessels (lymphangiogenesis) support cancer development and progression. Additionally, in the same way, nerves are able to provide a means for cancer to metastasize and invade new tissues. Here, nerves may provide a “pathway” along which cancer cells can migrate [13]. Nerves interact with multiple tissues throughout the body, through direct interaction or via chemokines, cytokines, hormones, and neurotransmitters. Tumour cells generally have receptors for these different molecules and can also secrete a wide array of them. This provides a means for the nervous system to interact with and promote tumour development [13]. 

This review will discuss the role that the microbiome plays in the development and progression of various cancers, through the effect of the microbiota on the nervous system.

## 2. Contribution of Microbes to Hallmarks of Cancer

**Figure 1:**
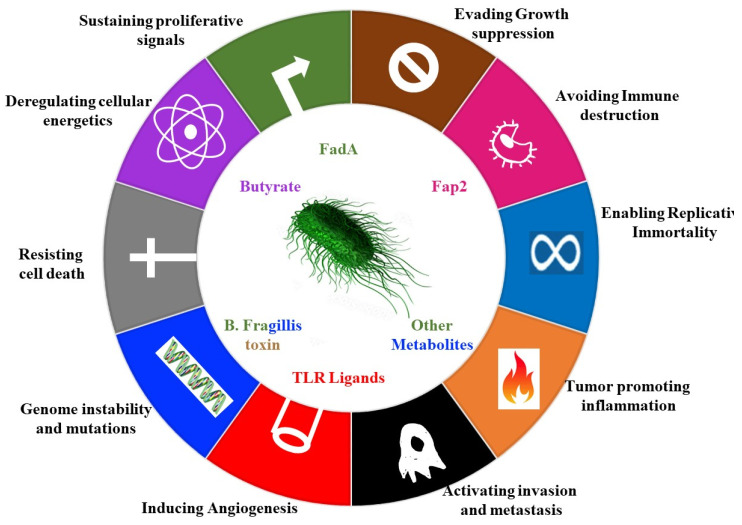
The effect of the microbiome on the hallmarks of cancer. This schematic represents the different effects that microbiota have within the body on different hallmarks of cancer. More specifically, the hallmarks of cancer are ten biological traits that define the transition from normal cells to cancer cells [26]. The bacteria can affect the induction of angiogenesis through secreting TLR ligands, deregulate cellular energetics through the secretion of the bacterial metabolite butyrate, alter immune system activation as well as inflammation, and initiate DNA damage and mutations.

Microbes act either directly or indirectly to facilitate development and progression of cancer. The notable example of direct action are gamaproteobaceria, which express the cytidine deaminase, which metabolizes and thus inactivates the chemotherapeutic gemcetabine [14]. Examples of indirect action include the induction of DNA damage through the production of mutagens and the promotion of inflammation by reactive oxygen species (Figure 1) [15].

The *pks+* strain of *E. coli* drives carcinogenic processes in a multitude of ways Including by induction of double-strand breaks, aneuploidy, cell-cycle arrest, and improper cellular division [16]. Mechanistically, the *E.coli pks+* bacteria invade the colonic epithelial cells using the carcinoembryonic antigen related cell adhesion molecule 6 (CEACAM6) receptor, and once inside the cell, they release the toxin pks+ [17].

Microbes can also stimulate cancer promoting signalling pathways, such as the e-cadherin– Wnt–b-catenin signalling pathways [16,18]. Enterotoxigenic *Bacteroides fragilis* (ETBF), secretes the *B. fragilis* toxin (BFT) (Table 1), which accelerates the endogenous cleavage of e-cadherin [19]. ß-catenin, normally bound to e-cadherins, is then liberated, and translocates to the nucleus to promote transcription of c-Myc, leading to epithelial cell proliferation (Figure 1) [19]. 

*Fusobacterium nucleatum* achieves a similar interaction with e-cadherin via its adhesin FadA (Table 1) [20]. Since the e-cadherin–β catenin complex regulates cellular adhesion, any interference with the complex can lead to loss of cellular adhesion, increased movement of tumour cells, invasion and metastasis [21].

Gut microbiota have been shown to promote obesity-linked liver cancer through metabolites and microbial components, such as lipoteichoic acid (LTA)—a Gram-positive gut microbial component. LTA promotes cancer by increasing the senescence-associated secretory phenotype (SASP) of the hepatic stellate cells (HSC), secreting inflammatory cytokines and growth factors. LTA also increases the expression of cyclo-oxygenase-2 (COX2), through Toll-Like Receptor-2 activation [22].

Complicit microbes are microbes that promote cancer but are unable to directly cause cancer. These are often involved through their production of bioactive metabolites that modulate immune function. For example, microbes associated with lung cancer can stimulate the expression of interleukin 1β (IL-1β) and interleukin-23 (IL-23), leading to inflammation and tumour cell proliferation [21]. Jin et al. used the *Sftpc-Cre;Kras*^LSL-G12D/+^;*p53*^fl/fl^ to drive the expression of Cre recombinase in lung epithelial cells, thereby inducing expression of oncogenic Kras^G12D^ and knock out of the tumor-suppressor gene *p53.* Jin et al. harvested the lungs from 8 and 15 weeks old mice maintained under two different conditions: the first group was placed under germ-free conditions and the second group was placed in specific-pathogen free conditions. Jin et al. then stained the lungs for the proliferation marker Ki67 and showed that the lungs from mice grown in specific-pathogen free conditions were more heavily proliferating (i.e., Ki67-positive) compared to the lungs of the germ-free grown mice, demonstrating that the presence of commensal bacteria in the specific pathogen free grown mice exacerbated the proliferation of tumor cells. When Jin et al. examined the bronchoalveolar lavage fluid (BALF) from all mice and submitted BALF to 16sRNA qPCR, they identified the genuses *Staphylococcus*, *Streptococcus*, *Lactobacillus* and *Pasteurellaceae.* Furthermore, the specific pathogen free mice had elevated levels of the cytokines Il-1β and Il-23 compared to the germ-free mice, indicating the commensal bacteria were stimulating cytokine production. Using FACS, they showed the GF mice had elevated numbers of γδT cells and the elevated γδT cells localized specifically in the lungs, where the γδT cells were responsible for producing the majority of the Il-17 pro-inflammatory cytokine. When the SP-free mice were treated with the UC7-13D, which acts against the γδT cells, the number of neutrophils was decreased, showing the elevated γδT cells promote neutrophil infiltration. Jin et al. argue the commensal bacteria can exacerbate tumors via stimulating the proliferation of γδT cells, which also produce pro-inflammatory cytokines like CXC2 and IL17 and promote neutrophils to infiltrate lungs [23].

Another example is the induction of angiogenesis (Figure 1). Pathological angiogenesis is augmented by microbial products such as lipopolysaccharide. These components activate pathways leading to angiogenesis by binding to Toll-like receptors [24]. Suppression or modulation of the immune system and the initiation of the inflammatory response also lead to increased angiogenesis as well as invasiveness [25].

## 3. Gut Brain Axis

**Figure 2:**
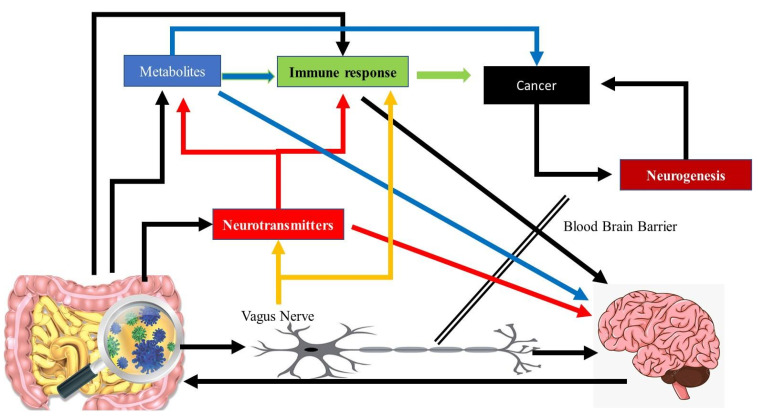
The Gut Brain axis: Schematic outlining the interactions between the gut–brain axis and cancer cells. The microbiota–gut–brain axis consists of the brain, glands, gut, immune cells, and gastrointestinal microbiota. Altered microbial populations can result in the development of cancer in multiple ways. These include the altered secretion of bacterial metabolites, the altered immune response due to the presence of different bacteria and the altered expression of neurotransmitters. These three factors can interact with each other to promote the development of cancer. The Vagus nerve connects the gut and the central nervous system. Increased Vagus nerve activity correlates with increased cancer progression and metastasis. The vagus nerve communicates with the gastrointestinal system through the release of neurotransmitters, excessive or lowered release of neurotransmitters, in response to changes in the microbiome. The selective blood–brain barrier regulates the transport of molecules such as neurotransmitters, or metabolites that are derived from the microbiota. This means the blood–brain barrier can regulate the influence of the microbiota upon the brain.

The correct composition of the populations of the microbiota is important for the maintenance of the correct homeostasis of any region of the body [27]. It has been well established from the way that an individual’s ‘emotional’ state can affect digestion, that the nervous system can play an important role on the gut and vice versa. This bidirectional interaction has been dubbed the gut–brain axis [28,29]. The microbiota–gut–brain axis consists of the brain, glands, gut, immune cells, and gastrointestinal microbiota (Figure 2) [30]. Both the central [31] and enteric nervous systems [32] regulate the communication between the gastrointestinal tract and the brain and apart from the nervous system, it is also regulated through hormones and immunological signalling [8,33]. Multiple lines of evidence confirm the existence of the gut–brain axis. Studies have shown that germ free (GF) animal models have altered cognitive function [34,35]. The behaviour of animals [36] and humans [37] can also be altered by feeding them specific strains of bacteria. The different populations of microbes in the human gut, have been classified into three different enterotypes, based on the dominant genus of the microbe present in each type: *Bacteroides, Prevotella*, and *Ruminococcus* [38]. Antibiotic treatment, influences the heterogeneity of microbiota, altering the activity of the enteric nervous system and the brain [39]. Additionally, radiotherapy is now known to affect the composition of the GI microbiome, leading to changes in the composition of metabolites produced and excreted by the microbiome [40]. Most importantly, the presence of certain microbial pathogens or the proportions of one species of bacteria in comparison to other bacteria in the gut can contribute to the etiopathogenesis of different types of cancer. Initially, changes in the composition of the microbiota were only known to be associated with the development of colon cancer. However, it is now known that changes in gut bacteria populations are related to not only gastrointestinal cancer but also prostate, pancreatic [41], blood (leukaemia) [42] and brain cancers [12]. 

### 3.1. Metabolites Released by Microbes

**Figure 3:**
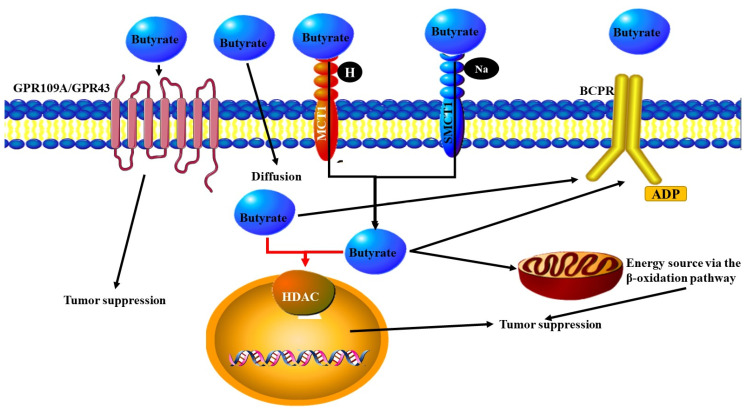
The function of the secondary microbial metabolite butyrate. This figure represents a summary of butyrate entry into the cell and the effects it has on gene expression and tumorigenesis. Butyrate is a Short Chain Fatty Acid (SCFA). High levels of Butyrate have neuropharmacological effects and influences the brain indirectly via regulating the immune system and vagus nerve activity. This metabolite is produced by many bacteria through fermentation. Butyrate can enter cells by binding to MCT1/SLC16A1; SMCT1/SLC5A8, GPR43/FFAR2; GPR41/FFAR3 and GPR109a/HCAR2 receptors. It can also actively diffuse through the cell membrane. Once in the cell butyrate can be used by tumor cells as an energy source via the β-oxidation pathway and it can also alter gene expression by inhibiting histone deacetylases (HDACs). This results in butyrate promoting apoptosis and inhibit cellular proliferation through epigenetic modifications.

One of the most important ways that the microbiota is able to influence both the nervous system and the contribution of the nervous system to the development of cancer is through the release of metabolites (Table 1) (Figure 1 and Figure 2). Commensal gut bacteria contribute to the development of colon cancer through the acceleration of DNA damage and induction of chromosomal instability. This occurs through the ability of the gut bacteria to trigger macrophages leading them to produce clastogenic agents [43]. Another important class of metabolites are the Short Chain Fatty Acids (SCFAs) that are produced as a result of the fermentation of non-digestible carbohydrates by anaerobic commensal bacteria. SCFAs have been found to function as energetic substrates for epithelial cells. Among the SCFAs one of the most important is butyrate. Other SCFAs include acetate, propionate and the structurally related ketone bodies (e.g., acetoacetate and d-β-hydroxybutyrate) [44]. High doses of butyrate have neuropharmacological effects by influencing the brain indirectly via regulating the immune system and vagus nerve activity. Butyric acid is produced by many bacteria (Figure 3) [44]. Butyrate and other SCFAs exert their influence by binding to specific receptors. These include MCT1/SLC16A1; SMCT1/SLC5A8; GPR43/FFAR2; GPR41/FFAR3 and GPR109a/HCAR2. Butyric acid can also be used by tumor cells as an energy source via the β-oxidation pathway [44]. Cancer cell metabolism relies on a process known as the Warburg effect. This describes the way in which cancer cells depend mainly on glucose as a carbon source using the glycolytic cycle. The use of butyrate as an energy source means that it alters the metabolic pathway favoured by the cell. Therefore, the use of butyrate in this way exerts an anticancer effect by starving cancer cells [45]. Additionally, when butyrate is not metabolized rapidly it remains at a high concentration intracellularly. The end result of this is inhibition of histone deacetylases (HDACs) [44], which results in the promotion of apoptosis and inhibition of cellular proliferation mediated by epigenetic modifications [45]. Colon cancer patients were found to have lower numbers of many butyrate-producing bacteria in their stool thus resulting in lower levels of butyrate. The bacterial species *Akkermansia muciniphila* was found at higher levels in the stool of colon cancer patients. These bacteria are able to degrade mucin, which may result in changes in the levels of metabolites present in or absorbed from the intestinal tract [46]. In another study, Singh et al. showed that Gpr109a had an important effect in colonic chronic inflammation and carcinogenesis through its dual role as a receptor of both niacin (a well-known vitamin) and butyrate. [47].

### 3.2. The Vagus Nerve

The Vagus nerve (VN) functions as an important communications channel between the gut and the central nervous system [48]. Retrospective studies have indicated that increased activity in the vagus nerve is observed as cancer progresses or metastasizes [49]. The vagus nerve communicates with the gastrointestinal system through the release of neurotransmitters in response to changes in the environment or changes brought about by factors such as infection by different pathogens and cancer [50]. Over or under activation of the vagus nerve therefore leads to excessive or lowered release of neurotransmitters, respectively, resulting in aberrant digestion, gastric motility or signalling along the brain gut axis [51]. The vagus nerve is also regulating immune function [52] as well as the rate and ease of absorption of substances from the intestine [53].

During the processing of ingested food, the enteroendocrine cells lining the gut secrete various factors to communicate with nearby neurons and the microbiota [54]. The microbes also release metabolites that bind to receptors on the surface of the enteroendocrine cells, thereby regulating their function. Some of the hormones produced by the enteroendocrine cells include serotonin or 5-hydroxytryptamine (5-HT), cholecystokinin (CCK), and peptide YY (PYY) [55]. These responding enteric neurons then communicate with the afferent fibres of the vagus nerve, which then conduct the signals to the brain [56].

### 3.3. Blood–Brain Barrier

The blood–brain barrier (BBB) is a selective barrier that allows for the transport of numerous specific molecules. These include signalling molecules, nutrients, and minerals. The specific signalling molecules that are allowed to cross from the circulatory system, include those that are transported from the gut to the brain. The barrier arises from the endothelial cells of the brain capillaries and consists of multiple layers of tight junctions, adherence junctions, membranes and uncharged small lipid soluble molecules [57]. Some of these molecules include proteins such as occludin and claudin. The BBB also helps prevent the free movement of cells and therefore acts as a barrier to brain metastasis of most tumour types. The BBB has consistently been an impedance to the treatment of brain cancers as it limits the access of drugs to the brain, as many chemotherapeutic agents do not cross the blood–brain barrier BBB. Some drugs may even weaken the BBB allowing more rapid and extensive metastasis [58]. Many of the metabolites secreted by the gut microbiome have similar characteristics to the molecules that are transported across the blood–brain barrier; and are therefore able to cross [57]. The integrity of the blood–brain barrier, however, is also depending on the microbiota and the molecules they secrete. For instance, in germ free mice the blood–brain barrier becomes more permeable as the microbiota, that normally assist in the expression of molecules to form tight junctions, are absent [59]. This situation can be reversed by inoculating these germ-free mice with bacteria like *Clostridium tyrobutyricum* which produce high levels of butyrate [59]. The BBB also allows for communication between the nervous and immune systems. The BBB regulate the passage of immune cells and several other factors associated with immunity [60].

### 3.4. The Gut Microbiome and the Immune System

Based on studies initially performed on the role played by altered gut microbiomes in prostate cancer, it was established that certain pathogenic gut bacteria are able to trigger an immune inflammatory response (Table 1), which promotes the development of prostate cancer [61]. Gut bacteria may help to stimulate the immune response to other types of cancer as well (Figure 2). Treatment of mouse models using the chemotherapy drug cyclophosphamide, resulted in certain Gram-positive bacteria to move into secondary lymphoid organs resulting in the immune system responding to these bacteria by producing T helper 17 (pTh17) cells and memory Th1 immune responses. This resulted in decreased levels of cancer and increased treatment success. This response was not observed in mice treated with antibiotics to kill Gram-positive bacteria. Tumours in these antibiotic treated mice were also resistant to cyclophosphamide treatment [62].

## 4. The Microbiota and the Immune System in Nerve Related Cancer

The microbes in the various tissues activate the immune system in multiple ways. Much of the communication between microbes and the immune system, however, depends on the lymphatic system. It was long thought that the brain did not possess a lymphatic system. However, a substantial and immunologically significant lymphatic drainage system has since been discovered. This system functions to drain lymphatics from brain to cervical lymph nodes [72]. This system, also known as the glymphatic system, is another means by which the nervous and immune systems can engage in bi-directional communication. This system connects the peripheral lymphatic tissues to the CNS [73]. The pseudo-lymphatic role of the glymphatic system implies that it also plays a role in neuroinflammation.

### 4.1. The Inflammatory Response

**Figure 4:**
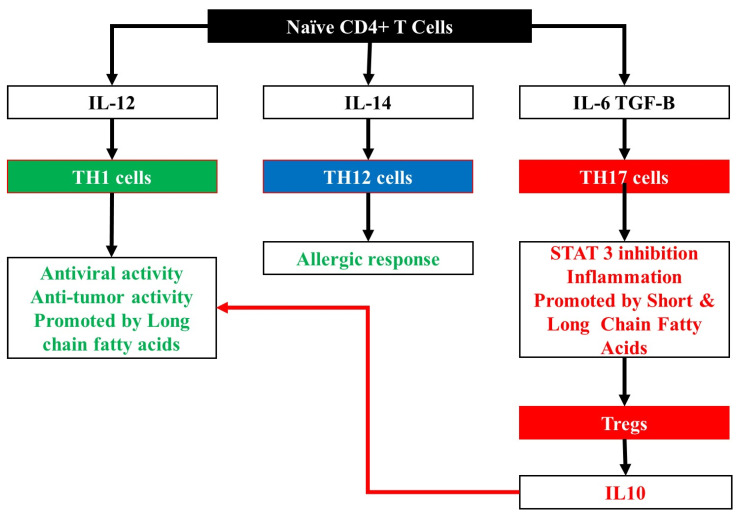
The differentiation of T-cells in response to inflammatory cytokines. The schematic above demonstrates the signals from the microbiome resulting in the differentiation of T-cells into specific sub-populations. The secretion of the IL-12 and IL-14 cytokines result in dendritic cells initiating the development of Th1 and Th2 cells, respectively. Th1 cells are vital for the immune system to mount an effective response against tumour cells. T-helper-17 (Th17) cells arise from IL-6 and TGF-B signalling pathways. These cells secrete pro-inflammatory cytokines.

The inflammasome is triggered by the innate immune system through the recognition of pathogen recognition receptors. Once the inflammasome is activated it recruits both the apoptosis-associated speck-like protein, which contains a caspase recruitment domain, and the cysteine protease caspase 1. The caspase 1 cleaves pro-IL-1β and pro-IL-18 to make active IL-1β and IL-18 proteins [74]. Different gut microbiome populations have been shown to activate the inflammasome to different extents. In addition, the activation of the inflammasome has been shown to have different neuronal effectors on mouse models, resulting in behavioral and cognitive changes [75]. Certain pro-inflammatory cytokines are known to be involved in the development of brain cancer. These include IL1β, IL6, IL8, IL12, GM-CSF, and TNFα [76]. Some commensal gut bacteria are able to assist dendritic cells to initiate the development of Th1 and pro-tumorigenic T-helper-17 (Th17) cells, leading them to secrete pro-inflammatory cytokines (Figure 4) [77,78] The contribution of the microbiome to the inflammatory response was further demonstrated by the fact that germ free mice have a decreased inflammatory response and a lower level of pro-inflammatory cytokines [79]. In lung cancer, T cells in the lung tissue, γδ T cells, react to the presence of microbiota in the lung and activate an inflammatory response. IL-1β and IL-23 are inducing proliferation and activation of the γδ T cells, which subsequently produce IL-17, which in turn promotes more proliferation [23]. Another pro-tumorigenic response is initiated by the bacteria *Fusobacterium nucleatum*. This bacterium dampens the anti-tumour immune response. It achieves this through the use of the Fap2 (Table 1) adhesion protein to inhibit the ability of cytotoxic immune cells to kill tumours. It is also able to stimulate the T-cell immune-receptor, immunoglobulin and immunoreceptor tyrosine-based inhibitory motif domains (TIGIT) which functions as an immune inhibitor [80]. Increased levels of *F*. *nucleatum* in the microbiome is associated with higher numbers of tumour-associated macrophages which function to inhibit antitumor T-cell responses [81].

Anti-cancer effects initiated by the immune response involving the microbiota, include the response initiated by the intestinal bacteria of the genus *Bifidobacterium*. These bacteria increase the tumour-killing capabilities of cytotoxic T cells by assisting the function of dendritic cells [82]. The cytotoxic T-lymphocyte-associated protein 4 (CTLA4) is a negative regulator of T cell responses. Therapies such as anti-CTLA4 antibodies, that block CTLA4 function have an anti-tumour effect. The efficacy of these therapies depends on the presence of the bacteria *Bacteroides thetaiotamicron* and *B. fragilis* [83]. The immune response initiated by the presence of polysaccharides secreted by *B. fragilis*, such as polysaccharide A (PSA), can enhance antitumor immune responses. This function is achieved through Toll-like receptor 4 (TLR4) (Table 1)– and IL-12–dependent TH1 responses. This suggests that the immune activation facilitated by the presence of these bacteria also initiates an antitumor response that is enhanced by CTLA4 inhibition [83]. 

### 4.2. Bacterial Metabolites and the Immune Response

One of the mechanisms by which bacteria can affect the immune response and either promote or inhibit cancer development is through the production and secretion of secondary metabolites. Once secreted into the gut they can enter the circulatory or lymphatic system and circulate throughout the body [84]. Some of these metabolites secreted by bacteria are CNS-related neurotransmitters and neuromodulators [85]. Others are the previously mentioned SCFAs [86]. SCFAs decrease the levels of pro-inflammatory cytokines that are released as part of an immune response, by affecting Th1 cell populations. SCFAs promote Treg development, which in turn secrete IL10 secretion/ IL-10 impairs the anti-cancer activity of Th1 cells and therefore promotes cancer development [86]. The presence of high concentrations of the bacteria *Bacteroides fragilis* leads to increased formation of Tregs that secrete IL-10 [87]. 

Long chain fatty acids are another type of metabolite released by microbes. These increase the proinflammatory response by increasing the rates of differentiation of T cells to form increased numbers of Th1 and Th17 cells. The expression of pro-inflammatory factors such as TNF-α, IFN-γ, and Csf2 is also increased [88]. 

### 4.3. NF-κB Signalling Pathway

**Figure 5:**
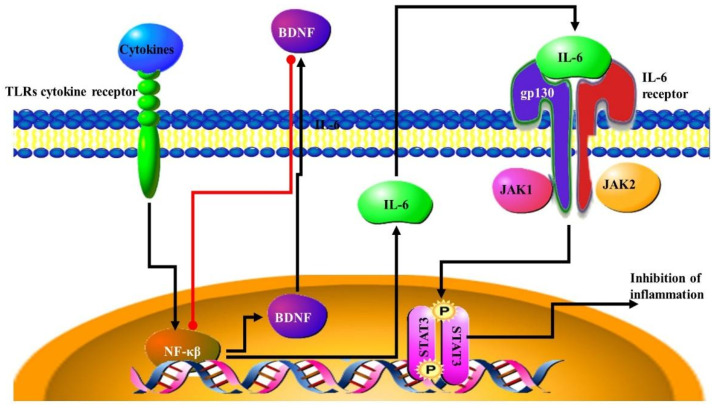
NF-KB and IFN1 signalling in immune responses: NF-κB signalling has been found to activate the immune system and result in the secretion of cytokines into the CNS, and inflammation in the CNS. Increasing NF-κB activity is also associated with the inhibition of brain-derived neurotrophic factor (BDNF). BDNF is important for the formation of new nervous tissue which assists in the development and progression of cancer. The NF-KB pathway is activated by cytokines such as TNF. In brain cancer, the STAT3 pathway is activated through the IL6- NF-κB signalling which results in an aggressive cancer.

NF-κB signalling has been found to activate the immune system in response to altered gut microbiome populations. For example, higher levels of *Campylobacter jejuni* in the gut led to the secretion of cytokines which lead to the activation of NF-κB [89]. NF-κB signalling in response to changes in the gut microbiota can lead to CNS inflammation. This was observed in the neurons of mice. This increase in NF-κB activity is also associated with the inhibition of brain-derived neurotrophic factor BDNF (BDNF) expression (Figure 5) [90]. BDNF is important for the formation of new nervous tissue which assists in the development and progression of cancer as new nerve fibers assist in the expansion and migration of tumours [91]. In brain cancer, the transcription factor STAT3 pathway is activated through the IL6-NFκB pathway, which results in aggressive cancer that progresses faster and a poor prognosis for the patient. This is a result of the suppression of the inflammatory response (Figure 5) [92,93]. The STAT3 pathway can be blocked by blocking IL-17 signalling, resulting in decreased inflammation and tumorigenesis [94].

Bacteria from the genus *Helicobacter* play an important role in both prostate and colon cancer. Many unique *Helicobacter* species were isolated exclusively from patients with gastrointestinal cancers [46]. Mice infected with the bacteria *Helicobacter hepaticus* were found to be more commonly affected by prostate intraepithelial neoplasia and microinvasive adenocarcinoma lesions, without the accompanied presence of IBD or large adenomatous polyps in their bowel. When lymphoid node cells were extracted from these mice and injected into healthy mice, the majority of these mice developed neoplasias. A high concentration of mast cells secreting TNF-α and proteases were present, leading to elevated NF-κB signaling in the prostate of the *Helicobacter hepaticus* infected mice. It was assumed that these mast cell secretions contributed to carcinogenesis [95].

### 4.4. Immune Cells in the CNS

Immune cells within the brain not only defend it against infection and injury, but also assist with processes such as neural remodeling and plasticity. Due to it being partially separated from the rest of the body by the blood–brain barrier, the central nervous system must have its own native immune cells. These include glial cells, macrophages, CD8+ T cells, Tregs, other CD4+ T helper (Th), microglia and astrocytes. These cells are involved in both the adaptive and innate immune systems [96]. Gut microbiota can release antigens that stimulate immune signalling pathways through CD4+ T cell activation and resulting in Th17 cell activation [97]. The SCFAs butyric acid and propionic acid produced by microbes discussed previously can cross ΒΒΒ, transported through the blood and have the potential to regulate T cell differentiation in other tissue sites as well. This activation was accompanied by increased expression of the transcription factor Foxp3 through altering the *foxp3* promoter activity [98]. Germ free mice have also been shown to have microglia with abnormal morphological characteristics. These microglia also have altered gene expression [99]. 

Microbial metabolites are able to activate astrocytes from their resting state. They achieve this by acting on aryl hydrocarbon receptors involved in IFN-I signalling, thereby restricting the recruitment and activity of neurotoxic immune cells to initiate anti-inflammatory activity [100]. In germ-free mice, microglia have altered structure and show higher levels of the receptors that are involved in the differentiation and proliferation of immune cells: colony stimulating factor 1 receptor (CSF1R), F4/80 and CD31, on their surfaces. These receptors are normally only found in high numbers on the surface of immature microglia cells. As microglia mature the expression of these receptors decrease. The activation of the GPR43 receptor on innate immune cells activates the inflammatory response. The same observations have been noted in mice that have been treated with antibiotics. In both germ free and antibiotic treated mice, the microglia numbers remain high [101]. Microglia from germ free mice also show increased expression of multiple genes, this increased gene expression is typical of younger microglia [102]. Germ free mice show defects in their microglia activity [100].

### 4.5. Type I Interferon Signalling Pathways

Type I interferon (IFN-I) is a cytokine induced by pathogen-associated molecular patterns (PAMPs) to prime the immune system to recognise various viral, bacterial and tumour cells. IFN-1 is also active in the CNS and is known to play a role in the protection against brain cancer in animal models [103], reviewed in [104]. IFN-I is associated with the maturation of dendritic cells and cytotoxic T cells, which are both involved in the immune response against cancer cells [105]. IFN-I also exerts an anti-cancer activity through its ability to regulate growth and induce apoptosis in haematological cancers [106]. IFN-1 expression can affect or be affected by the microbiome [107]. TLR3 can be activated by increased numbers of lactic acid bacteria in the gut. Once activated TLR3 increases the secretion of INF-β secretion from dendritic cells [108]. 

## 5. Neurotransmitters in Cancer and the Microbiome

Receptors for neurotransmitters are commonly expressed on the surface of tumour cells. These include receptors such the G protein coupled receptors (GPCRs), otherwise known as serpentine receptors. Once neurotransmitters bind to these receptors they can alter the behaviour and characteristics of tumour cells. This can result in increased proliferation, migration, and a more aggressive tumour [109]. Tumours can also produce and secrete neurotransmitters. An example of this is that prostate cancer cells behave like neuroendocrine cells in their ability to secrete neurotransmitters. This response is increased in tumour cells that have been exposed to therapeutic agents and the cells may have done this in response to these agents [110]. 

The monoamine neurotransmitter, Serotonin or 5-hydroxytryptamine (5-HT) is able to act on the central nervous system (CNS), neuroendocrine system (enteric nervous system) [111,112] and the immune system [113]. It is known that serotonin interacts with the microbiome and plays a role in the development and progression of various cancers [114]. Contradictory to this, lower levels of serotonin may also promote colon cancer development, as low levels of serotonin are accompanied by increased levels of DNA damage, increased inflammation, and consequently increased levels of CRC development [115]. The production of much of the serotonin within the body is regulated by the gut microbiota. The enterochromaffin cells located in the gut supply serotonin to the mucosa, lumen, and circulating platelets and these cells are stimulated to produce serotonin through the actions of spore-forming bacteria [112]. Germ free male mice were also found to have higher levels of serotonin in their hippocampi. This is preceded by an increase in the tryptophan in the blood of the male rats, which is the precursor of serotonin [116].

In addition, serotonin stimulates proliferation in various cancers such as gliomas (where it also plays a role in migration) [117], prostate cancer [118], bladder cancer [119], small cell lung carcinoma [120], colon cancer [121], breast cancer [122], and hepatocellular carcinoma [123]. One of the processes affected by serotonin that contributes to cancer development and progression is angiogenesis. Increased serotonin leads to increased blood vessel development and an increase in the size of blood vessels [124,125]. Studies have also focused on the use of altered serotonin or serotonin receptor [126] expression patterns as a diagnostic or prognostic biomarker in various cancers including urological cancers [126], and colon cancer [127]. The receptors most commonly associated with the development and progression of cancers are the 5-HT_1_ and 5-HT_2_ receptors [128,129,130]. Activation of these receptors alters the cell cycle progress, stimulates cell growth and results in improved cell viability this is due to the activation of genes such as MEK-ERK1/2 and JAK2-STAT3 signalling pathways [124]. Increased expression of these receptors has been identified in ovarian [131], and prostate cancer [132]. In some cases, antagonists of serotonin receptors, inhibitors of selective serotonin transporter and of serotonin synthesis have been successfully used to prevent cancer cell growth in prostate cancer [133]. 

Importantly, microbiota-dependent effects on gut 5-HT significantly impact host physiology, modulating gastrointestinal motility and platelet function. Metabolites from spore forming bacteria were isolated in higher amounts from the feces of patients with high colonic and blood levels of 5-HT, suggesting that gut microbes signal directly to neuroendocrine cells. This was further demonstrated by the fact that in germ free mice higher concentrations of certain metabolites increases the level of 5-HT in the colon and blood. Spore forming bacteria are therefore able to control 5-HT levels in the host [112].

### 5.1. Catecholamines, Norepinephrine and Dopamine

The migration of cancer cells was found to be stimulated by neurobiological signals, namely through the signalling of norepinephrine [134]. The correct levels of the neurotransmitter may depend on the correct populations of bacteria within the gut as germ free mice have significantly lower levels of norepinephrine [135]. In addition to dopamine stimulating dopaminergic neurons, they activate the innate and adaptive immune cells [136]. The implications of the activation of the immune systems in the development of cancer has already been discussed. Dopamine is also synthesized and secreted by various bacteria [137].

### 5.2. Gamma-Aminobutyric Acid (GABA)

Bacteria from the genera *Lactobacillus* and *Bifidobacterium* are able to produce the neurotransmitter gamma-aminobutyric acid (GABA) [138]. GABA binds to the serpentine receptor GABA(B) and the signal is internalised through a decrease in the level of the cyclic AMP [138]. GABA was found to reduce the migration of colon cancer cells in culture through modulating the activity of norepinephrine [134]. 

### 5.3. Acetylcholine

The neurotransmitter acetylcholine has been found to play a role in many different cancers. It induces cell growth and division in epithelial cells [139], and the increased expression of acetylcholine receptors has been identified in multiple cancer types in mouse models including acetylcholine receptor 3 (M3R3) in gastric cancer [140] and the acetylcholine receptor M (Chrm1) muscarinic receptors in prostate cancer on stromal cells [141]. *Lactobacillus* subspecies can produce acetylcholine [137]. Ganglia in both the sympathetic nervous system (SNS), consisting of ganglia which are parallel to the spinal cord, and the parasympathetic nervous system (PNS), consisting of the vagus nerve and some spinal nerves, respond to acetylcholine stimulation. However, only the PNS produces and secretes it (reviewed in [142]). This is important as the vagus nerve is one of the major connections between the brain and the gut microbiota. 

## 6. Neurogenesis and miRNA Regulation by Microbiota

The creation of new nervous tissue (neurogenesis) is an important process for the progression of most cancers. Tumour cells produce factors that lead to the formation of new nerve tissue [143]. These newly formed nerves release neurotransmitters that stimulate tumour growth and migration [144]. Cancers can invade new tissue and migrate along nerves or nerve tissue. Like angiogenesis and lymphogenesis these new nerves also support the new tumour leading to the growth of cancers around these new nerves in a process known as perineural invasion (PNI) [145]. The microbiome is also able to initiate signalling cascades which stimulate neurogenesis by activating TLR2. The process of neurogenesis can be inhibited, delayed, or even counteracted by feeding animals a mixture of specific bacteria that changes the populations of their gut microbiota [146,147].

The regulation of gene expression through the actions of miRNA is known to play a role in neuronal proliferation, neurogenesis, and Brain Derived Neurotrophic Factor (BDNF) signalling. These processes as well as the expression of certain miRNAs is altered in germ free mice [148]. Studies involving the next generation sequencing of miRNA from normal, germ free and antibiotic treated mice indicate that miRNA expression in the amygdala and prefrontal cortex is regulated by the microbiota and changes in the microbiota populations result in changes in the expression of miRNA. The miRNA expression pattern of germ-free mice was altered once again following bacterial colonisation of the germ-free mice [149], One of the miRNAs whose expression is targeted by gut microbiota is miR-206-3p. This miRNA is known to regulate the expression of the neurotrophic BDNF [149,150]. BDNF is known to stimulate growth of neurons and is important for the cancer related neurogenesis that is also involved in the invasion, metastasis and support of cancer development and growth (Reviewed in [142]).

## 7. Treatments for Cancers Based on the Microbiome Neural Interactions

Inoculation of patients with specific commensal microbiota are now known to have beneficial effects in a variety of cancers [151,152]. For example, when the supplementation of the diet of mice with bacteria of the genus Bifidobacterium is part of a treatment strategy that also include PD-L1 blockade, it augments the anti-cancer growth inhibition elicited by PD-L1. This occurs through pathways that initiate the maturation of dendritic cells, those that stimulate tumour specific CD8+ T cells, signals that recruit immune cells and the activation of IFN 1 pathways [82]. The bacteria *Bifidobacterium longum* had an inhibitory effect on the development and progression of colon cancer. Studies have shown that the use of *B. longum* supplements on colon cancer suppressed colon cancer incidence. It is now known that these bacteria inhibit azoxymethane-induced cell proliferation as well as lower the activity of onco-proteins such as ras-p21 and ornithine decarboxylase [153]. 

However, there is a problem concerning the use of microbial inoculation as a treatment method for cancer. Traditional treatment such as chemotherapy and radiotherapy may have negative impacts on the microbial population. In addition to this, the use of antibiotics may also disturb the microbiota (both those that are already present as well as those that have been given to a patient as a treatment). This has been demonstrated by treating mice with tumours with the immunostimulatory drug, cyclophosphamide. When combined with antibiotics the drug was far less effective in treating the cancers. This was due to the lower levels of Th1 and Th17 cells [62]. 

In addition to these therapeutic techniques involving microbiota and the nervous system function in cancer, research has been conducted on the use of microbiota to lessen the side-effects of cancer treatment. Following chemotherapy, it is common for patients to experience post-chemotherapy abdominal pain. This pain seems to be as a result of microbial toxicity leading to changes in the microbial effects on nerves capable of sensing pain. A study has reported that this pain can be lessened through probiotic treatment of the patient [154]. This can reconstitute the microbiota that was lost following chemotherapeutic treatment [155]. Another complication of chemotherapy is known as chemotherapy-induced cognitive impairment (CICI). This disorder involves decreased memory, attention, and concentration as a result of chemotherapy and is related to cytotoxic effects on the CNS. It may also be compounded by neuroinflammation and BBB damage. Once again this is thought to be due to chemotherapy disrupting the gastrointestinal microbiota. Research has been conducted to show that probiotic supplementation of the microbiome can assist in treating CICI [12]. 

The change in the population of microbes may be used as a diagnostic tool [27]. Since the changes in the microbiome may be cancer specific [156], the changes may be used as a personalized diagnostic tool. This can be exploited by examining transcriptome or proteome profiles of cancer patients. Whole transcriptome or proteome analysis has been used to detect cancer specific pattern changes [157]. However, there are several problems using microbial populations as diagnostic biomarkers. Firstly, the microbial biomass is much lower than that of the host while secondly there is a high risk of contamination by the environment and other microbes not isolated from the patient. 

## 8. Conclusions

**Figure 6:**
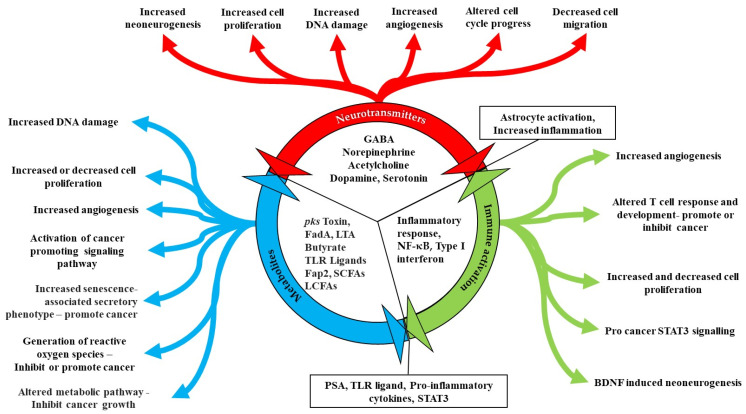
Schematic illustrating the Nerve related contribution of the Microbiome to the development of cancer. The microbiome can influence the synthesis of neurotransmitters as well as some microorganisms having the ability to synthesize neurotransmitters of their own. This is related to the secretion of specific metabolites by the microorganisms making the microbiome that have the ability to promote or inhibit cancer in various ways. The presence of different microorganisms can also alter the immune specific response to these microorganisms. All these responses can be mediated through the specific response of the nervous system to the presence of neurotransmitters, metabolites, and the activation of the immune system.

The concept of the microbiome affecting the development and progression of cancer through interactions involving nerves, neurotransmitters, the immune system, and metabolites secreted by microorganisms (Figure 6) is most clearly seen in the example of the gut brain axis. However, this interaction occurs throughout the body and is influenced not only by the ability of the gut microbiome to release metabolites that can stimulate or inhibit nervous function, but also through the microbiome influencing the immune system and the production of cytokines resulting in altered neural function. These relationships are currently being investigated for their ability to provide future therapeutic targets, through the use of probiotics to alter the microbiota in a patient’s body and thereby increase the levels of certain microbial ‘species which excrete metabolites with an anti-tumour function. Additionally, these microbes may activate the immune response allowing a greater number of anti-tumour immune cells to be created. The changes in the populations of microbes in patients with various cancers are also being explored as new diagnostic or prognostic biomarkers. 

## Figures and Tables

**Table 1:** Important bacteria and their influence on cancer progression.

Metabolites			
Anti-Cancer			
Organism	Activity	Mechanism	Ref
*Enterococcus faecalis*	Produces extracellular superoxide	Cell damaging ROS	[63]
*Helicobacter pylori* or *Bacteroides fragilis*	Activate the host’s spermine oxidase, which, in turn, promotes gastric cancer	Generates hydrogen peroxide and reactive oxygen species	[64]
*Lactobacillus casei*	Ferrochrome	Trigger apoptosis in tumor cells via JNK pathway	[65]
Bacteria of the genus *Propionibacteria*	Butyrate, propionate	Inhibit histone deacetylase	[66]
Pro-oncogenic			
Organism	Activity	Mechanism	Ref
*Akkermansia muciniphila*	Degrade mucin leading to changes in the levels of metabolites	Higher levels in colon cancer	[67]
*Enerococcus faecalis*	Superoxide	ROS production	[66]
*B. acteroides* *fragilis*	MP toxin	B catenin pathway activation ROS production	[66]
*Clostridium coccoides*	Β-glucuronidase-	Deconjugates liver-catabolized and plant-derived estrogen’s, enabling them to bind and activate the estrogen receptors expressed by target cells	[68]
*Clostridium leptum*	Β-glucuronidase-	Deconjugates liver-catabolized and plant-derived estrogen’s, enabling them to bind and activate the estrogen receptors expressed by target cells	[68]
*Escherichia coli*	Colibactin and cytolethal distending toxin (CDT)	Generate DNA double-strand breaks genomic mutations	[68]
*Fusobacterium nucleatum*	FadA	Amplify tumorigenesis through E-cadherin–Wnt–β-catenin signaling	[18]
*Helicobacter pylori*	CagA	p53 degradation, B catenin MAPK, AKT pathway activation, ROS production	[66]
*Salmonella strains*	AvrA	Amplify tumorigenesis through E-cadherin–Wnt–β-catenin signaling	[20]
*Salmonella enterico*	AvrA	B catenin MAPK AKT pathway activation	[66]
*Shigella flexneri*	IpgD VirA	p53 degradation	[66]
Immune related activity			
Organism	Activity	Mechanism	Ref
*Clostridium orbiscindens*	TLR3 signaling	TLR3-mediated INF-β secretion by DCs in the intestine	[69]
*Lactobacillus acidophilus*	TLR2 signaling	Anti-viral responses via TLR2-dependent IFN-β in murine bone marrow-derived DCs	[70]
*Salmonella enterica*	Mon phosphoryl lipid A (MPL)	Adjuvant in anti-cancer vaccines	[66]
*Fusobacterium nucleatum*	bacterial virulence factor Fap2, able to bind and block the NK inhibitory receptor	Inhibits host’s Natural Killer (NK) cells stimulating cancer formation by blocking immune effectors that normally inhibit tumorigenesis	[68]
*Porphyromonas gingivalis*	Activation of TLR4 to produce increased levels of cytokines	Stimulates astrocytes contributes to the formation of neuroinflammatory lesions	[71]
